# Variations and influential factors of the seed oxygen isotope ratios across 42 forest plant species

**DOI:** 10.1007/s00442-026-06007-z

**Published:** 2026-11-11

**Authors:** Shoji Naoe, Koichiro Sawakami, Yoshihiro Tsunamoto, Takashi F. Haraguchi, Ichiro Tayasu

**Affiliations:** 1 Tohoku Research Center, Forestry and Forest Products Research Institute, Morioka, Iwate Japan; 2 Nikko Botanical Garden, Graduate School of Science, University of Tokyo, Nikko, Japan; 3 Research Institute for Humanity and Nature, Kita-Ku, Kyoto, Japan; 4 Present Address: Research Institute of Energy, Environment and Geology, Hokkaido Research Organization, Sapporo, Hokkaido Japan; 5 Present Address: Biodiversity Research Center, Research Institute of Environment, Agriculture and Fisheries, Osaka Prefecture, Neyagawa, Osaka Japan

**Keywords:** Botanical garden, Isotope, Life form, Precipitation, Seed, Temperature

## Abstract

Oxygen isotope ratios (^18^O/^16^O, hereafter *δ*^18^O) of plant tissues are widely used for various purposes, including paleoclimatic reconstruction, identification of the geographical origin of plant-based products, and understanding plant water use. On the other hand, *δ*^18^O values of seeds have been rarely measured. We measured the *δ*^18^O values of whole seed testas of 42 forest plant species at a single location to clarify the variation in *δ*^18^O values of seeds among species and its influential factors for exploring how they can be used. The maximum interspecific difference in *δ*^18^O values of seeds was 16.7‰, which is comparable to the values reported in those of plant tissues collected worldwide. This large variation is influenced by the climate and life form. Precipitation during the month of seed maturation and one to two months beforehand had a negative effect on the *δ*^18^O values of seeds. The *δ*^18^O values of seeds varied among life forms, that is, canopy and subcanopy trees > shrubs ≥ lianas. This may reflect vertical differences in water uptake or transpiration due to variations in root depth or plant height. Our results demonstrate that the *δ*^18^O values of seeds of wild plants contain detectable climatic and ecological signals, particularly those related to precipitation and life forms. These findings suggest that the *δ*^18^O values of seeds provide a useful basis for future studies aiming to reconstruct past environmental conditions and investigate water use during seed production.

**Supplementary Information:**

The online version contains supplementary material available at https://doi.org/10.1007/s00442-026-06007-z.

## Introduction

Oxygen isotope ratios (*δ*^18^O) of plant tissues are widely used for various purposes. Their typical examples are paleoclimatic reconstruction in climatology, identification of the geographical origin of plant-based products in forensic science, and understanding the plant water use in ecophysiology and crop science (Barbour 2007; Sternberg 2009; Carter and Chesson 2017). These studies are based on the fact that the *δ*^18^O values of plant tissues are determined by two main phases: plant-absorbed precipitation, whose *δ*^18^O values depend initially on climatic and geographic factors (e.g., precipitation, temperature, and elevation), and subsequent processes after water absorption, which are driven by both environmental and biotic factors—such as evaporation from leaves, diffusion during transpiration, and isotopic exchange between organic molecules and plant water (Bowen and Wilkinson 2002; Dawson et al. 2002; Barbour et al. 2005; Treydte et al. 2014). For example, the summer climate of the past 2,600 years can be extracted from the *δ*^18^O values of tree ring cellulose, which are determined by precipitation (Nakatsuka et al. 2020). The worldwide isoscape (i.e., isotopic distributions across landscapes) of the *δ*^18^O values of cotton cellulose, which was estimated based on the isoscape of the *δ*^18^O values of precipitation, can be a tool for tracking the source of counterfeit money composed of cotton (West et al. 2010a). The uptake depth of water by plants can be estimated by using the *δ*^18^O values of xylem water and those of potential water resources represented by soil water, which derive from precipitation (Bachofen et al. 2024). Tree rings, leaves, and xylem water have been widely used materials in the previous studies of the *δ*^18^O values of plant tissues. Regarding the *δ*^18^O values of seeds, while they have not received much attention, research on cultivated crops represented by rice, wheat, coffee beans has become increasingly active in recent years (e.g., Carter and Chesson 2017; Cueni et al. 2022a). These studies revealed that the *δ*^18^O values of seeds/fruits can be higher than (Driscoll et al. 2020; Cueni et al. 2022b), lower than (Cueni et al. 2022b), or equivalent to (Sanchez-Bragado et al. 2019), those of leaf and stem depending on plant species, that the *δ*^18^O values of seeds/fruits can be used to identify the geographical origin of rice, wheat, coffee beans and bell peppers (Carter and Chesson 2017; Driscoll et al. 2020). While available research remains limited, the *δ*^18^O values of seeds show varying climate responses: whole rice correlates positively with temperature (Akamatsu et al. 2014) and negatively with relative humidity (Kaushal and Ghosh 2018) in alignment with established trends in other plant tissues (Barbour 2007), whereas coffee bean water exhibits the exact opposite correlations (Driscoll et al. 2020). On the other hand, there has been very little research on wild plants, which are more directly exposed to natural influences, leaving fundamental information about potential usefulness of the *δ*^18^O values of seeds lacking.

To date, the *δ*^18^O values of seeds of wild plants have been studied from the viewpoint of seed dispersal (i.e., identification of mother plants of dispersed seeds) (Naoe et al. 2016a, 2019). These studies found a negative correlation between elevation and the *δ*^18^O values of non-dispersed seeds of several woody species. The correlation is used to estimate the elevational locations of mother plants of dispersed seeds, thereby evaluating the elevational seed dispersal. Considering that the *δ*^18^O values of plant tissues are governed by climate more so than simple geography through the *δ*^18^O values of precipitation (Carter and Chesson 2017), the negative correlation suggests elevation-related climate effects, such as an increase in precipitation and a decrease in temperature with elevation, both are known to decrease the *δ*^18^O values of precipitation (Dansgaard 1964; Rozanski et al. 1993; Terzer-Wassmuth et al. 2021). In addition, annual variations in the *δ*^18^O values of seeds in the same woody species suggest the effect of annual variations in past climate (Naoe et al. 2016b; Tsunamoto et al. 2025).

If the *δ*^18^O values of seeds from wild plants reflect climate, they may be useful for more than just identifying locations of mother plants from dispersed seeds. For example, the *δ*^18^O values of seeds that is available from seed bank, archaeological remaining and museum collection (Gerrienne et al. 2004) may be useful in reconstructing past climates, just like tree rings. Compared to tree rings, seeds have the advantage that samples can also be obtained from herbaceous plants and that the samples potentially have a higher temporal resolution of climate information because they are formed over a shorter period of time. A correlation between the *δ*^18^O values of seeds and the fruiting dates across woody species in Japanese temperate forests, where climate vary seasonally, suggests that the *δ*^18^O values of seeds reflects seasonality of climate (Naoe et al. 2016b). In fact, climatic factors such as temperature and relative humidity before seed maturation affects the *δ*^18^O values of the cultivated rice (Akamatsu et al. 2014; Kaushal and Ghosh 2018) and strawberry fruits (Cueni et al. 2021). In addition to reconstructing past climates, it may be possible to determine when and what water resources plants use during seed production by examining the *δ*^18^O values of seeds. However, at present, only a limited number of plant species (ca. 10 species including crops) have been measured for the *δ*^18^O values of seeds (Carter and Chesson 2017; Naoe et al. 2025), and the overall image of the *δ*^18^O values of seeds remain unclear. Especially, basic knowledge is limited; how much variation exists in the *δ*^18^O values of seeds among plant species, how much of this variation can be explained by plant traits, and whether the *δ*^18^O values of seeds generally reflect climate.

The aim of this study was to clarify the species-specific and environmental effects on the *δ*^18^O values of seeds. To this aim, we measured the *δ*^18^O values of seeds of various forest plant species at and around a botanical garden, where the environment is fairly uniform. We targeted whole seed testa, composed primarily of cellulose and lignin (i.e., similar to woody material such as tree rings). We use these data to answer the following questions: 1) How much variation exists in the *δ*^18^O values of seeds among forest plant species at a single location?, 2) Do climate and plant traits affect the *δ*^18^O values of seeds?

## Materials and methods

### Study site

The study was conducted at the Nikko Botanical Garden and its surrounding area in the Tochigi Prefecture, central Japan (36°45’N, 139°35’E, 630–660 m a.s.l.). The garden is the institute for research and education of the University of Tokyo and has rich collections of temperate and alpine plants of Japan and adjacent regions. Tree crowns generally cover the garden, and plants in the garden receive water from precipitation (i.e., no irrigation). The garden belongs to the cool-temperate zone. The annual mean temperature and precipitation are 11.7 ºC and 2,159 mm, respectively, according to the data from a meteorological station at the garden (Shimizu and Ayabe 2016). Seasonal variations in climatic conditions such as mean temperature and precipitation during the survey years (2015, 2016) are shown in Figure S1. Highest and lowest mean temperatures were recorded in August and January (22.1 and 0.5ºC), and highest and lowest precipitation were recorded in September and December (779 and 4 mm) in the study years.

### Sampling

We visited the Nikko Botanical Garden on August 25th, October 9th and 22nd in 2015, June 29th and August 23rd, September 21st, October 21st in 2016, and collected seeds from the fruiting plants at the time. We collected seeds from one fruiting individual per species, considering that plant size, age, and varieties are unlikely to affect the *δ*^18^O values of seeds in conspecific species (Cueni et al. 2022a; Naoe et al. 2025; Naoe et al., unpublished data) and that the environment in the garden is fairly uniform. As a result, we were able to collect seeds from 42 forest plant species in 25 families, which differ greatly in ecological characteristics such as fruiting dates and life forms (Table S1). Plant nomenclature followed Yonekura & Kajita (2021).

### Isotope analysis

For isotope analysis, three seeds per species were used except for *Taxus cuspidata var. nara*, for which we could collect only one seed. We used the most external dead and woody tissue of a seed (i.e., testa, which surrounds the embryo and nutritive tissues) as the analysis subject (Naoe et al. 2016b). This is because the *δ*^18^O values of live tissues may show diurnal variations (ref. Cernusak et al. 2002), and because the *δ*^18^O values of testa, of which growth and differentiation proceed coordinately with the development of the endosperm and embryo (Debeaujon et al. 2007), are likely to represent the isotopic information of the seed sufficiently. As a result, we analyzed anatomically various tissues including exocarp, endocarp and seed coat depending on the species. We used raw materials without cellulose extraction (i.e., bulk) for efficiency assuming that they have sufficient isotopic information (Barbour et al. 2001; Gori et al. 2013; Lee et al. 2015). While prior studies on the *δ*^18^O values of seeds have often relied on bulk analysis as in this study (e.g., Naoe et al. 2016b; Kaushal and Ghosh 2018), the biochemical composition of seeds—specifically the proportions of cellulose and lignin—can be species-dependent. Because oxygen isotope ratios vary between these components (Gray and Thompson 1977; Barbour et al. 2001), interspecific comparisons based on bulk tissue must be interpreted with caution (see Discussion for details). Each seed tissue was ground to a weight of approximately 0.13 mg and placed into silver capsules (Säntis Analytical, Milan, Italy), which were rolled into balls. The *δ*^18^O values of seeds were determined using a high-temperature elemental analyzer (TC/EA, Thermo Fisher Scientific, Waltham, Massachusetts, USA) connected online to an isotopic ratio mass spectrometer (IRMS) (Delta plus XP; Thermo Fisher Scientific) via a ConFlo III interface (Thermo Fisher Scientific). To prevent potential moisture contamination in the samples, molecular water absorbed from the atmosphere was removed by a drying oven at 60 °C for at least one night before measurements. Oxygen isotope analyses were performed by measuring the CO produced through high-temperature carbothermic reduction of the endocarp at 1400 °C in the presence of excess carbon. Before entering the mass spectrometer, the helium stream containing CO was separated from H_2_ and N_2_ using a 1.4-m molecular sieve 5A chromatographic column maintained at 90 °C. The sample gases were calibrated by analyzing reference substances (IAEA-601 and 602 benzoic acid, and USGS-54 and 56 whole wood: 23.14‰, 71.28‰, 17.79‰, and 27.23‰, respectively) with known isotope compositions (Brand et al. 2009; Hunsinger et al. 2010; Qi et al. 2016). The isotopic composition of a sample is conventionally expressed as a “*δ*” value per mill (‰) compared to international primary reference material (Vienna Standard Mean Ocean Water, V-SMOW) using the following formula:

$${\delta }^{18}O = {R}_{sample} / {R}_{reference} - 1$$

where *R* denotes the ratio of numbers (*N*) of each isotope, as follows:

$$R = N{(}^{18}O) / N{(}^{16}O)$$

The standard deviation of the replicates was approximately 0.2‰ (1σ) for measurements.

### Statistical analysis

To explore the factors influencing the *δ*^18^O values of seeds, a linear mixed-effects model (generally, abbreviated as LMM or LME) with an identity link and a gaussian error was employed, using the “lmer” function in the “lme4” package in R (Bates et al. 2023). The *δ*^18^O values of seeds served as the response variable. We considered mean temperature, precipitation amount, mean vapor pressure deficit (VPD) and relative humidity during seed maturation (details provided later), dates of flowering and fruiting (elapsed days from January 1st, maximum of 365), life forms (canopy tree, subcanopy tree, shrub, liana, and herb), and leaf longevity (i.e., evergreen or deciduous) as explanatory variables, with plant family and species as random factors to control the phylogenetic effect. Monthly mean temperature and precipitation were calculated using hourly data available from the meteorological station at the botanical garden maintained by garden staffs. Monthly mean VPD and relative humidity were calculated using hourly data available from the Okunikko meteorological station maintained by Japan Meteorological Agency, which is 8 km away from the botanical garden (Japan Meteorological Agency 2025). Flowering and fruiting dates were generally measured based on field observation or the information available on the website of the botanical garden (Nikko Botanical Garden 2025). For species we could not get flowering and fruiting dates at the botanical garden, we used the dates measured in a cool-temperate temperate forest (Ogawa Forest Reserve) in the same Kanto region with similar latitude and elevation (36°56’N, 140°35’E, ca. 650 m a.s.l.) (Naoe unpublished data; Shibata et al. 1998). Life form and leaf longevity of each species was determined referring the literatures (Ohashi et al. 2015, 2016a, b, 2017a, b).

The model selection was carried out through the following three processes. First, we identified which months’ climatic conditions had the greatest influence on seed oxygen isotope ratios, we created models that included climatic conditions in either the month of seed maturation or one, two, three, four, five, or six months prior to the month of seed maturation, while leaving other explanatory variables unchanged. Multicollinearity among the explanatory variables was assessed using an adjusted generalized variance inflation factor (aGVIF) (Fox and Monette 1992), using the “vif” function in the “car” package in R (Fox and Weisberg 2019). We omitted relative humidity which are highly correlated with VPD (e.g., the Pearson correlation *r* for relative humidity and VPD in the previous month of the seed-maturation month was −0.93) to avoid multicollinearity. We also omitted dates of fruiting which are highly correlated with precipitation (e.g., *r* for precipitation in the previous month of the seed-maturation month and dates of fruiting was 0.71). In addition, we omitted dates of flowering, as these are unlikely to affect the *δ*^18^O values of seeds directly, and there was indeed only a weak correlation between them (*r* = −0.27). As a result, all explanatory variables tested in the following analysis exhibited aGVIF values of < 3.16 (equivalent to the conventional VIF threshold of 10.0) with two exceptional models which are statistically inferior to the best models (Table S2, S3), suggesting that multicollinearity was not a major concern. The fitted models were compared based on corrected Akaike’s information criterion (AICc) (Sugiura 1978), and models with ∆AICc > 2 were considered different (McCullagh and Nelder 1989). As a result, the model incorporating climatic conditions one month prior to the seed-maturation month was selected as best model with no competitive model (Table S2). Second, we identified the length of the time window of the past climate which most influenced the *δ*^18^O values of seeds. Considering that climatic conditions one month prior to the seed-maturation month was selected as most influential in the above analysis, we built models incorporating mean climatic conditions during 0–1, 1, 1–2, 1–3, 1–4, 1–5, 1–6, 0–2, 0–3, 0–4, 0–5, and 0–6 months prior to the seed-maturation month (as for precipitation, we used sum, not mean), while leaving other explanatory variables unchanged. As a result, the models incorporating climatic conditions during 1–3, 1–4, 1–5, 0–2, and 0–3 months prior to the seed-maturation month was selected as best or competitive models based on AICc (Table S3). Third, we regarded each best or competitive model as full model and further compared and hierarchically ranked the models with all combinations (subsets) of explanatory variables in the full model based on AICc by using “dredge” function in the “MuMIn” package in R. As a result, the model including precipitation during 0–2 months prior to the seed-maturation month was selected as best model with no competitive model (Table 1). To calculate the variance explained by the explanatory variables of the AICc best model, the marginal Nakagawa’s *R*^*2*^, which is suited for LMM, was adopted by using “r2” function in the “performance” package in R (Nakagawa and Schielzeth 2013). Moreover, to evaluate the relative importance of each explanatory variable included in the best model, we calculated partial Nakagawa’s *R*^*2*^ (the decrease in marginal Nakagawa’s *R*^*2*^; ∆*R*^*2*^) by removing each variable from the best model. All analyses were conducted using R 4.5.1. (R Development Core Team 2025).

**Table 1:** Corrected Akaike’s information criteria (AICc) best and null linear mixed-effects models (LMMs) to predict the oxygen isotope ratios of seeds, with species and family considered as random factorsk

	Coefficient						SD of the random effect PNG		AICc	ΔAICc
	Intercept	Precipitation	Mean Temperature	VPD	Leaf longevity	Life form	Species	Family		
best model	27.7241 (< 0.001)	-0.0064(< 0.001)	–	–	–	+(< 0.05)	1.7752	1.3638	449.3	
null model	19.9057 (< 0.001)	–	–	–	–	–	2.5797	1.9957	473.2	23.9

The coefficients of the models are shown with *P* values in parentheses, As for climatic conditions (i.e., precipitation, mean temperature and VPD), the values during the month of seed maturation and one to two months beforehand were used

## Results

### Variations of the seed oxygen isotope ratios across 42 forest plant species

**Fig. 1:**
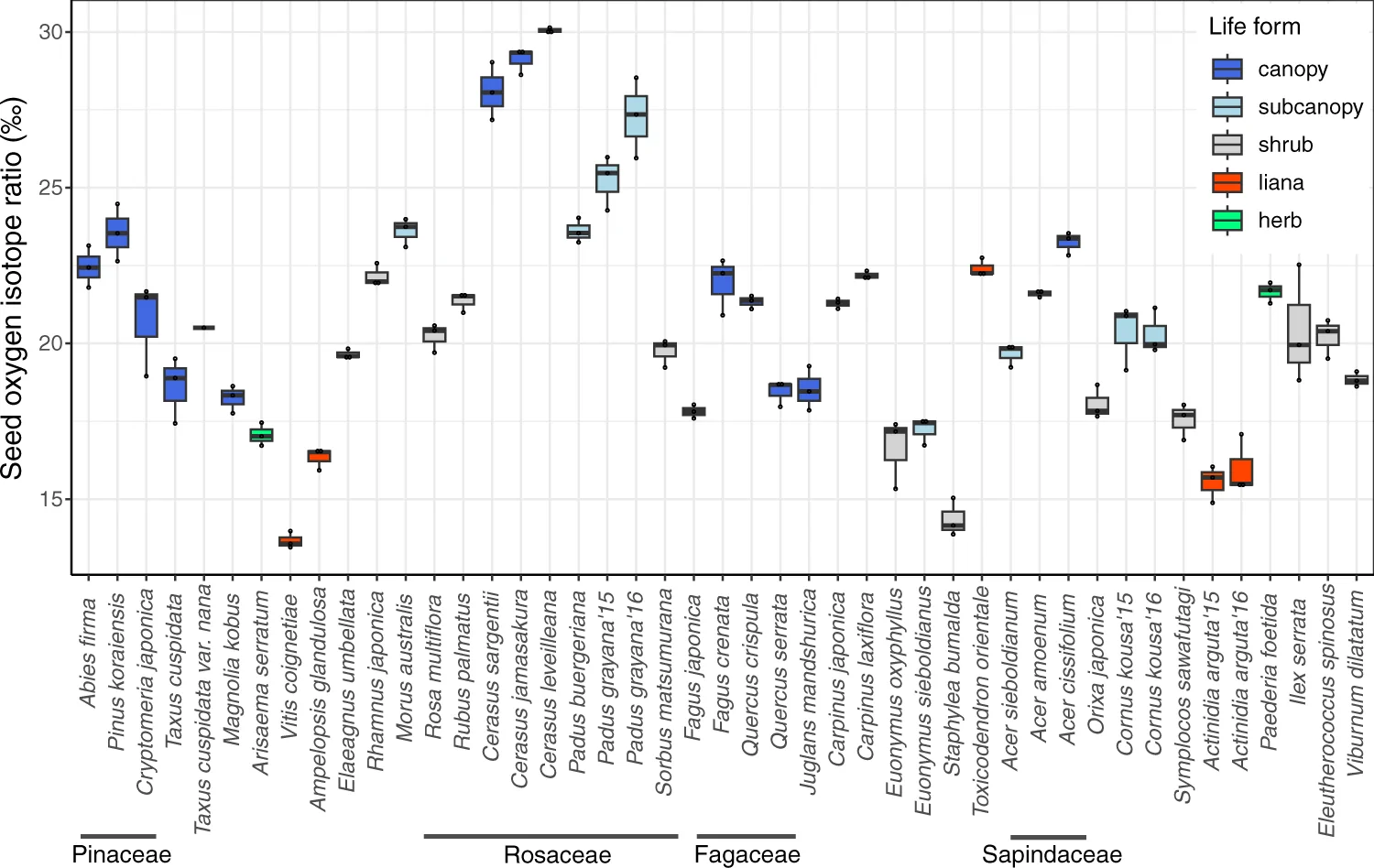
Variations of the seed oxygen isotope ratios across 42 forest plant species. The species are ordered phylogenetically. Families for which seed oxygen isotope ratios have been measured for three or more species are indicated in the figure. The sample size is three per species except *Taxus cuspidata var. nara*, for which we could collect only one seed. The sampling was conducted during the daytime at the Nikko Botanical Garden on August 25th, October 9th and 22nd in 2015, June 29th and August 23rd, September 21st, October 21st in 2016. As for *Padus grayana*, *Cornus kousa* and *Actinidia arguta*, the seed oxygen isotope ratios in 2015 and 2016 are shown

Large variations were observed in the *δ*^18^O values of seeds among species (Fig. 1). The minimum *δ*^18^O values of seeds was 13.5‰ of *Vitis coignetiae*, and the maximum value was 30.1‰ of *Cerasus leveilleana*. Intraspecific differences in the *δ*^18^O values of seeds were much smaller than the interspecific differences, and the maximum intraspecific difference (4.3‰) was observed in *Padus grayana* sampled in 2015 and 2016. The *δ*^18^O values of seeds tended to be similar within the same family of species. When looking at the same family, species in the same genus sometimes tended to have more similar values (i.e., *Cerasus* and *Padus* in Rosaceae), but in the other case this was not the case (*Fagus* and *Quercus* in Fagaceae).

### Factors influencing the seed oxygen isotope ratios

**Fig. 2:**
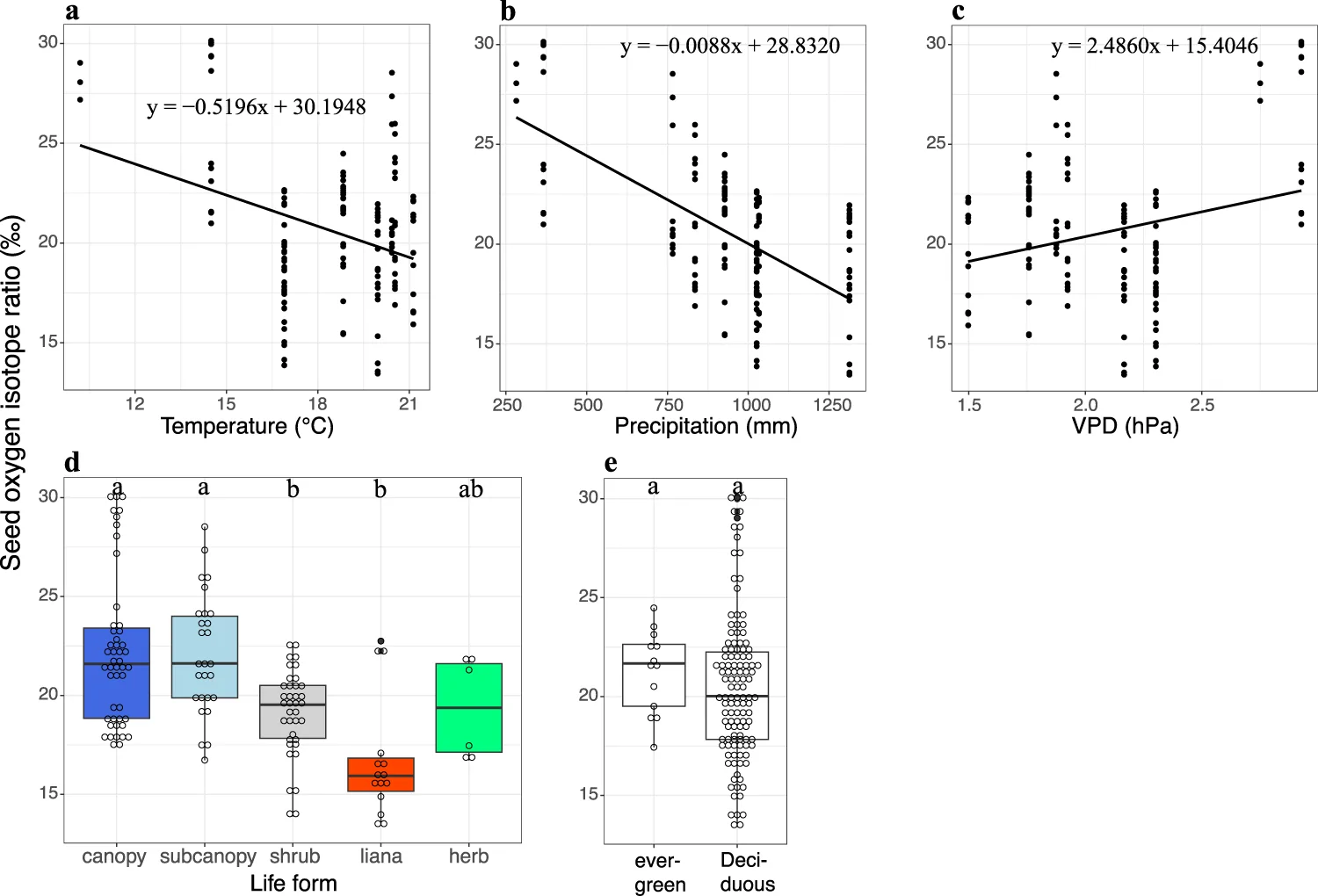
Simple correlation between the seed oxygen isotope ratios and the (**a**) mean temperature and (**b**) precipitation (**c**) VPD during the month of seed maturation and one to two months beforehand (**d**) life forms (canopy tree, subcanopy tree, shrub, liana, and herb), and (**e**) leaf longevity (evergreen or deciduous). In (**d**), (**e**), different alphabets indicate significant difference among groups (*P* < 0.05)

Simple correlations between the *δ*^18^O values of seeds and each variable are shown in Fig. 2. The *δ*^18^O values of seeds significantly decreased with respect to the temperature during 0–2 months prior to the seed-maturation month (Fig. 2a, R^*2*^ = 0.1092, *P* < 0.001). The *δ*^18^O values of seeds significantly decreased with respect to the precipitation during 0–2 months prior to the seed-maturation month (Fig. 2b, R^*2*^ = 0.3953, *P* < 0.001). The *δ*^18^O values of seeds significantly increased with respect to the VPD during 0–2 months prior to the seed-maturation month (Fig. 2c, R^*2*^ = 0.0678, *P* < 0.01). The *δ*^18^O values of seeds of ​shrubs and lianas were significantly smaller than those of canopy and subcanopy trees (Fig. 2d, Wilcoxon rank sum test corrected by Holm method, *P* < 0.01) and the *δ*^18^O values of seeds of ​lianas were tended to be smaller than those of shrubs (*P* = 0.0680). There was no significant difference between deciduous and evergreen species (Fig. 2e, Wilcoxon rank sum test, *W* = 953, *P* = 0.1912).

In the LMM analysis, the model that included precipitation during 0–2 months prior to the seed-maturation month, and life form was selected as the best model (Table 1). In the best model, the precipitation, and life form were significant variables. The higher the precipitation, the lower the *δ*^18^O values of seeds (Fig. S2). Regarding life form, the *δ*^18^O values of seeds of shrubs and lianas was lower than that of canopy trees. Nakagawa’s *R*^*2*^ of the best model was 0.490. Removing the precipitation from the best model reduced Nakagawa’s *R*^*2*^ to 0.293 (∆*R*^*2*^ = 0.197), while removing the life form reduced it 0.337 (∆*R*^*2*^ = 0.153). These results indicate that although both variables possess substantial explanatory power for the variance of the *δ*^18^O values of seeds, the precipitation makes a larger unique contribution to the model’s overall explanatory power.

## Discussion

The maximum interspecific difference in *δ*^18^O values of seeds was 16.7‰ (the range was 13.5–30.1‰), which was larger than the maximum intraspecific differences in *δ*^18^O values of seeds sampled near the lower and upper limits of the species distributions of six woody species reported so far (ca. 3 to 13‰) (Naoe et al. 2016b, 2025). The value was comparable to the maximum interspecific difference in *δ*^18^O values of leaf cellulose among 30 marine, freshwater, and terrestrial plant species collected from North and Central America (18.9‰, the range is 14.2–33.1‰) (Epstein et al. 1977). It was also comparable to the maximum interspecific difference in *δ*^18^O values of leaf water (18.8‰) and cellulose (14.5‰) in 192 terrestrial plant species comprised of 17 plant families within the eudicot clade (the ranges of leaf water and cellulose are 5.4–24.2‰ and 19.3–33.8‰, respectively) (Baan et al. 2023). We analyzed the whole testa as seed, and the testa is composed mainly of cellulose and lignin, similar to wood (Aspinall et al. 1967; Kawamura 1967; Gray and Thompson 1977). Thus, the *δ*^18^O values of these polymer compounds should determine the *δ*^18^O values of seeds. In fact, the *δ*^18^O values of the seeds (i.e., whole testa) and wood cellulose in Japanese larch *Larix kaempferi* were suggested to be similar (Naoe et al. 2025). The differences in the composition of cellulose and lignin among plant species might result in the observed interspecific differences in the *δ*^18^O values of seeds, because the *δ*^18^O values of cellulose and lignin are different (Gray and Thompson 1977; Barbour et al. 2001). The reported difference in *δ*^18^O values between wood cellulose and lignin of the same plant species is about 5–11‰ and that the *δ*^18^O values of whole wood are intermediate between the values of cellulose and lignin (Gray and Thompson 1977; Barbour et al. 2001). Therefore, it is suggested that up to approximately 5‰ of the 16.7‰ variation observed among plant species in this study can be attributed to this difference. Considering that the targeted plant species were collected from a single location and that they all inhabit at the cool-temperate forests in Japan, the magnitude of this variation—probably more than 10‰ even after removing the effect of seed biochemical composition among plant species—is remarkable and suggests that it may be useful for a variety of research. In the following sections, we primarily discuss the interpretation and usefulness of the climatic and ecological factors, which explained approximately half of the variation in the *δ*^18^O values of seeds.

We could identify the effect of precipitation and life form as factors influencing the *δ*^18^O values of seeds across 42 forest plant species, even though we analyzed the whole seeds of which tissue (i.e., testa) differed anatomically among species. These factors explained ca. 50% of the variation in the *δ*^18^O values of seeds (i.e., Nakagawa’s *R*^*2*^ = 0.490). Although negative effect of temperature and positive effect of VPD on the *δ*^18^O values were detected in each simple regression, their effects were not detected in LMM including other covariates. The *δ*^18^O values of precipitation, which are the basis for the *δ*^18^O values of plant tissues, are influenced by both climatic and geographical factors (Bowen and Wilkinson 2002; Yamanaka et al. 2015; Terzer-Wassmuth et al. 2021; Nelson et al. 2021). Because this study was conducted at a single location, the *δ*^18^O values of precipitation are determined by climatic factors. Climatic factors were considered to influence the *δ*^18^O values of seeds by reflecting seasonal changes in the *δ*^18^O values of precipitation, which is strongly influenced by precipitation (or its relatives such as relative humidity and VPD) and temperature (Terzer-Wassmuth et al. 2021; Nelson et al. 2021). In general, the *δ*^18^O values of precipitation decreases with increasing precipitation and with decreasing temperature (Dansgaard 1964; Rozanski et al. 1993; Terzer-Wassmuth et al. 2021), and thus it was expected that the *δ*^18^O values of seeds would follow the same pattern. In this study, the *δ*^18^O values of seeds decreased with increasing precipitation, which supported the prediction. Dansgaard (1964) demonstrated that the precipitation amount effect is fundamentally governed by a Rayleigh distillation process. During intense or prolonged precipitation events, the progressive rainout of an air mass preferentially removes heavier isotopes, leaving the remaining atmospheric moisture increasingly depleted. Consequently, the integrated precipitation from larger events exhibits lower isotope ratios. In the present study, seasonal variation in precipitation is likely to have influenced the *δ*^18^O values of seeds through changes in the *δ*^18^O values of precipitation. The seasonal variation in the *δ*^18^O values of precipitation was approximately 10‰ over a 23-year period at a city located about 30 km from the study site (Yabusaki et al. 2016), and this magnitude of variation could plausibly account for a substantial proportion of the 16.7% variation observed in the *δ*^18^O values of seeds. Precipitation might influence seed *δ*^18^O values not only through variation in the *δ*^18^O values of precipitation but also by constraining the evaporative isotopic enrichment of leaf water. Cheesman and Cernusak (2017) examined the *δ*^18^O values of leaf cellulose in several Eucalyptus species across a precipitation gradient of 235–1,388 mm in Australia, which was comparable to that observed in the present study (Fig. 2b). The study reported a variation of approximately 12.4‰ in leaf cellulose δ^1^⁸O values and concluded that this variation was attributable not only to differences in the *δ*^18^O values of precipitation but also to differences in evaporative isotopic enrichment of leaf water associated with precipitation. Similarly, precipitation-induced variation in leaf-water evaporative isotopic enrichment may partly explain the variation in the *δ*^18^O values of seeds observed in the present study. On the other hand, the effect of temperature on the *δ*^18^O values of seeds was not detected. The prevailing effect of precipitation compared to temperature was also reported in the studies of* δ*^18^O values of tree ring cellulose conducted at mid-latitudes (e.g., Nakatsuka et al. 2020).

The *δ*^18^O values of seeds varied among life forms; canopy and subcanopy trees > shrubs ≥ lianas. LMM analysis revealed that the unique effect of life forms on the *δ*^18^O values of seeds was smaller than, but of a magnitude comparable to, that of precipitation. There are two possible reasons for the differences among life forms, which are not mutually exclusive. First, this may be due to the difference in root depth across life forms. Root depth in the same habitat is roughly proportional to the plant size of the aboveground, and the order of deepest roots is as follows: canopy trees, subcanopy trees, shrubs, lianas, and herbaceous plants (Karizumi 2010; Fan et al. 2017). For example, when focusing on the several target plant species, the maximum root depths of canopy tree *Abies firma*, subcanopy tree *Morus australis*, shrub *Taxus cuspidata var. nana*, and liana *Vitis coignetiae* have been reported to be approximately 400, 190, 120, and 60 cm, respectively (Karizumi 2010). In the same cool-temperate forest ca.130 km away from the study site, it has been reported that the deeper the soil, the higher the *δ*^18^O value of the soil water, and the *δ*^18^O value of the groundwater is even higher (Tsujimura and Tanaka 1998). Larger plant species have deeper roots and therefore utilize soil water from deep soils or groundwater, with higher *δ*^18^O values, which could result in the higher *δ*^18^O values of their seeds. However, it should be noted that, although the sample size for herbaceous plants is small (N = 6), the increase in the *δ*^18^O value of soil water with increasing soil depth does not explain why the *δ*^18^O values of seeds of herbaceous plants with the shallowest roots was not statistically lower than that of canopy trees with the deepest roots. In the aforementioned study by Tsujimura and Tanaka (1998), the shallowest soil water sample was taken at a depth of 10 cm, and no soil water samples were taken at depths very close to the ground surface. Considering that the *δ*^18^O value of soil water is high near the ground surface due to active evaporation (e.g., Yabusaki and Tase 2007), herbaceous plants might also utilize soil water near the ground surface to produce seeds. In addition to absorbing water through their roots, plants absorb water from fog through their leaves, and this affects the *δ*^18^O value of leaf water (Goldsmith et al. 2017). It has been reported that the *δ*^18^O value of conifer leaf water tends to be higher than that of broad-leaved trees, shrubs, and herbaceous plants, due to water absorption from fog (Lehmann et al. 2020). The phenomenon observed in leaf water may also be observed in seeds. However, there was no difference in the *δ*^18^O values of seeds when comparing evergreen plants, which were all conifers, and deciduous plants (i.e., non-conifers) (Fig. 2d). Therefore, it seems unlikely that water absorption from fog contributed to the difference in the *δ*^18^O values of seeds among life forms. Second, this may be attributed to the vertical difference of the intra-forest microclimate, specifically VPD, driven by Japan’s humid precipitation regime and the closed canopy structure at the study site. Even within a humid climate, VPD at the forest canopy is higher compared to that at understory because of higher temperatures and atmospheric turbulence there (de Freitas and Enright 1995; von Arx et al. 2012; Vinod et al. 2022). Because higher VPD cause *δ*^18^O enrichment in leaf water and carbohydrates (Lehmann et al. 2017), higher VPD at the canopy could explain the higher *δ*^18^O values of seeds in canopy/subcanopy trees. In contrast, the forest understory buffers shrubs from harsh conditions through canopy shading and wind reduction. This effect, combined with soil moisture recharged by abundant precipitation, might maintains low VPD and high humidity, thereby minimizing evaporative isotopic enrichment and preserving lower *δ*^18^O values of seeds in shrubs. If this is true, it complicates the interpretation that plant species growing side by side necessarily experience the same climatic conditions, as the plant vertical structure may matter significantly. The *δ*^18^O values of seeds in canopy/subcanopy trees might be more appropriate for reconstruction of past climates in closed forests.

Our study revealed that the *δ*^18^O values of seeds vary widely among plant species, and that this variation is influenced by the climate and life form. The results indicate that the *δ*^18^O values of seeds of wild plants may provide a useful basis for reconstructing past climates. Reconstruction of past climates using *δ*^18^O values has mainly been carried out using tree rings and ice cores (West et al. 2010b). While a single seed cannot provide multi-year time series of oxygen isotopic information like tree rings or ice cores, seeds have properties that can compensate for the shortcomings of these materials. First, because seeds are produced over a relatively short time period, they can provide climate information with high temporal resolution more directly than tree rings, which generally require additional analytical procedures, such as intra-annual sampling or laser-ablation analyses, to achieve comparable temporal resolution (Esper et al. 2010; Miyahara and Locosselli 2024). In contrast to tree rings, seeds can provide climate information during the period of “non-active” tree growth, because seeds of non-tropical trees are commonly produced in autumn and winter (Naoe 2025). Second, the influence of climate was detected in cross-species analysis, indicating that it may be possible to reconstruct past climates at various periods of the year by using the seed oxygen isotope ratios of multiple plant species with different timing of seed maturation. But it is notable that mixing species would likely obscure climatic signals because interspecific variation in the *δ*^18^O values of seeds is still large even considering the effect of precipitation. To reduce the interspecific variation, the methodology should be further refined, for example restricting analyses to species within the same life form. Third, seeds can be obtained not only from trees but also from shrubs and herbs. This indicates the possibility of reconstructing past climates even in areas where trees cannot inhabit due to human disturbance or harsh environments such as polar circles and alpine zones where meteorological observations are difficult but drastic changes in vegetation and ecosystems due to climate change are of concern there (Post et al. 2019; Fazlioglu and Wan 2021). Fourth, the Juvenile effect, that is, the effect of tree age and size on the *δ*^18^O values of tree rings (Esper et al. 2010; Nakatsuka et al. 2020), is reported to be negligible in seeds because only adult plants produce seeds (Naoe et al. 2025). Last, seeds are easy to preserve and identify. Seeds remain in the ground for a long time (up to 385 million years) and can be identified to species with their morphology (e.g., Gerrienne et al. 2004). Biochemical compositions of seeds (testa, in this case) are similar to those of tree rings (Aspinall et al. 1967; Kawamura 1967; Gray and Thompson 1977), and thus they are expected to retain past climate information as oxygen isotope ratios for more than several thousand years, as with tree rings (Nakatsuka et al. 2020).

In addition to reconstructing past climates, *δ*^18^O values of seeds may provide useful information for plant ecophysiology and archaeology. Seed production and its annual fluctuations (i.e., masting) strongly influence plant regeneration and ecological interactions (Koenig 2021; Qiu et al. 2023; Bogdziewicz et al. 2024), and identifying the water resources used for seed development could improve our understanding of these processes. Such information may become increasingly important as precipitation regimes change under climate change (Shiogama et al. 2022). In archaeology, seeds are frequently preserved at sites, and their isotope compositions have been used to investigate past human activities (James et al. 2025; Styring et al. 2025). *δ*^18^O values of seeds may additionally help determine the geographic origin of plant resources and reconstruct patterns of collection or trade.

This study revealed that *δ*^18^O values of seeds vary greatly among plant species and that climate and plant traits influence the variation, demonstrating that *δ*^18^O values of seeds may be utilized in various research fields; however, this study has several important issues that must be improved in the future. We detected the effects of precipitation during the month of seed maturation and one to two months beforehand on the *δ*^18^O values of seeds across species. However, some species require more time to go from flowers to seeds, while some require less. It is necessary to focus on individual plant species and identify which period of past climates and the length of the time window most influenced the *δ*^18^O values of seeds. While we explained approximately 50% of the variation in the *δ*^18^O values of seeds among 42 forest plant species through precipitation and life forms, we could not investigate the detailed mechanisms how these factors influenced the *δ*^18^O values of seeds. This is a clear shortcoming of our study. Precipitation was considered to affect the *δ*^18^O values of seeds through the *δ*^18^O values of precipitation and evaporative isotopic enrichment of leaf water. However, testing this hypothesis requires quantitative assessment of the *δ*^18^O values of both precipitation and leaf water at the study site. We believe that the effects of life form on the *δ*^18^O values of seeds were mediated by differences in the δ^1^⁸O value of source water associated with root depth, as well as by differences in the δ^1^⁸O values of leaf water and carbohydrates associated with plant height. For sufficient verification of the former factors, it was necessary to examine the *δ*^18^O values of soil water and xylem water at the study site. A previous study in the same Japanese cool-temperate forest reported that the difference in *δ*^18^O values of water can used by plants was at most 1‰ at a certain time (Tsujimura and Tanaka 1998), which is considerably smaller than the difference between life forms (up to 5‰ on average). The *δ*^18^O values of precipitation and soil water change seasonally in Japanese cool-temperate forests (Hiiragi et al. 2025). Considering these factors, future study should investigate the *δ*^18^O value of soil water at different soil depths at a study site, as well as the seasonal changes in the *δ*^18^O values ratios of soil water and xylem water, to distinguish their respective effects and clarify their relative importance. Furthermore, to evaluate the impact of plant height via microclimate on the *δ*^18^O values of seeds, it is necessary to examine the oxygen isotope ratios of leaves. Remarkably, the relationship between the *δ*^18^O values of leaf water and cellulose can be weak, possibly because the influence of biochemical kinetic isotope fractionation is stronger than the influence of the *δ*^18^O values from precipitation (Baan et al. 2023). In seeds, too, the study of biochemical kinetic fractionation will be necessary to clarify the detailed mechanism of their *δ*^18^O values. Given that only about 50% of the substantial 16.7‰ variation in the *δ*^18^O values of seeds could be explained in the present study, future work should focus on extracting well-characterized compounds such as cellulose from seeds and determining their oxygen isotope composition to allow a more detailed evaluation of the mechanisms underlying this variation.

## Supplementary Information

Below is the link to the electronic supplementary material.

Supplementary file1 (DOCX 1071 KB)

## Data Availability

The datasets generated during the study are available from the corresponding author on reasonable request.

## Code availability

The R code used to analyze data in the current study is available from the corresponding author on reasonable request.
