# Supplementary material for: Variations and influential factors of the seed oxygen isotope ratios across 42 forest plant species
Source: Oecologia. 2026 Nov 11;208(12):170. doi: 10.1007/s00442-026-06007-z (PMC13754995; doi:10.1007/s00442-026-06007-z)
Supplement: Supplementary file 1 [file 442_2026_6007_MOESM1_ESM.docx]

**Variations and influential factors of the seed oxygen isotope ratios across 42 forest plant species**

*Shoji Naoe^1^, Koichiro Sawakami^2^, Yoshihiro Tsunamoto^1†^, Takashi F. Haraguchi^3‡^, and Ichiro Tayasu^3^

^1^Tohoku Research Center, Forestry and Forest Products Research Institute, Morioka, Iwate, Japan.

^2^Nikko Botanical Garden, Graduate School of Science, University of Tokyo, Nikko, Japan.

^3^Research Institute for Humanity and Nature, Kita-ku, Kyoto, Japan.

*Corresponding author. Email: disperser316@gmail.com, Tel.: +81–19–648–3942, Fax: +81–19–641–6747

†Present address. Research Institute of Energy, Environment and Geology, Hokkaido Research Organization, Sapporo, Hokkaido, Japan.

‡Present address. Biodiversity Research Center, Research Institute of Environment, Agriculture and Fisheries, Osaka Prefecture, Neyagawa, Osaka, Japan.

Table S1. Ecological characteristics of 42 forest plant species.

Table S2. Corrected Akaike's information criteria (AICc) of linear mixed-effects models (LMMs) that included mean temperature, precipitation, and mean vapor pressure deficit (VPD) in either the fruiting month or one, two, three, four, five, or six months prior to the fruiting month, while leaving other explanatory variables unchanged (i.e., life forms and leaf longevity), with species and family considered as random factors.

Table S3. Corrected Akaike's information criteria (AICc) of linear mixed-effects models (LMMs) that included mean temperature, precipitation, and mean vapor pressure deficit (VPD) in either during 0-1, 1, 1-2, 1-3, 1-4, 1-5, 1-6, 0-2, 0-3, 0-4, 0-5, and 0-6 months prior to the fruiting month, while leaving other explanatory variables unchanged (i.e., life forms and leaf longevity), with species and family considered as random factors.

Fig. S1. Seasonal variations in (a) mean temperature, (b) precipitation amount, (c) VPD, and (d) relative humidity in 2015 and 2016.

Fig. S2. Relationship between the seed oxygen isotope ratios and precipitation during the month of seed maturation and one to two months beforehand. Each colored solid line was estimated by LMM for each life form. Each colored area around each solid line indicates a 95% confidence interval.
